# Aggregation State of Residual *α*-Helices and Their Influence on Physical Properties of *S. c. ricini* Native Fiber

**DOI:** 10.3390/molecules24203741

**Published:** 2019-10-17

**Authors:** Kelvin O. Moseti, Taiyo Yoshioka, Tsunenori Kameda, Yasumoto Nakazawa

**Affiliations:** 1 Department of Biotechnology and Life Science, Graduate School of Engineering, Tokyo University of Agriculture and Technology, 2-24-16 Naka-cho, Koganei, Tokyo 184-8588, Japan; kelvinmoseti@gmail.com; 2 Silk Materials Research Unit, Institute of Agrobiological Sciences, National Agriculture and Food Research Organization, 1-2 Owashi, Tsukuba, Ibaraki 305-8634, Japan; yoshiokat@affrc.go.jp; 3 National Sericulture Research Centre, Industrial Crops Research Institute, Kenya Agricultural and Livestock Research Organization, P.O. Box 7816-01000 Thika, Kenya

**Keywords:** *S. c. ricini* silk, polyalanine sequence, *α*-helix, liquid fibroin, *α*-helix to *β*-sheet transition

## Abstract

Formation of the *α*-helical conformation in the poly-l-alanine (PA) sequence regions, subsequent structural transition to *β*-sheet during natural spinning, and presence of residual *α*-helices in *Samia cynthia ricini* (*S. c. ricini*) native silk fiber have been experimentally proven. However, the aggregation state of the residual *α*-helices, and their influence on the mechanical deformation behavior in native fiber remain unclear. Here we show that the *α*-helices form an ordered aggregation state with a hexagonal packing in the aqueous solution, some of which remain during natural spinning. X-ray scattering and differential scanning calorimetry (DSC) analyses revealed occurrence of a structural transition of the residual α-helices to the *β*-sheet structure, accompanied by disappearance of the plateau region in the force-strain curve, due to heat-treatment at ~220 °C. On the basis of X-ray scattering before and after tensile stretching of *S. c. ricini* native silk, a direct connection between the plateau region and the *α*-helix to *β*-sheet structural transition was confirmed. Our findings demonstrate the importance of the PA sequence regions in fiber structure formation and their influence on the tensile deformation behavior of *S. c. ricini* silk, features believed to be essentially similar in other saturniid silks. We strongly believe the residual ordered *α*-helices to be strategically and systematically designed by *S. c. ricini* silkworms to impart flexibility in native silk fiber. We anticipate that these knowledge forms a basis for fruitful strategies in the design and development of amino acid sequences for artificial silks with desired mechanical properties.

## 1. Introduction

Towards establishing a sustainable society, effective utilization of *Saturniidae* wild-silkworm silks is receiving increased attention because of their excellent material properties and abundant yields in nature [1]. Among them, silk produced by the semi-domesticated wild-silkworm, *Samia cynthia ricini* (*S. c. ricini*), is one of the most promising candidates. Its structure and mechanical properties have been well investigated, that is, the silk is one of the most studied among those obtained from non-mulberry sericulture [1,2]. Indeed, *S. c. ricini* silk fibroin (SF) is attracting increasing attention in diverse fields including the biomedical field as a source of biomaterials [3,4].

Earlier X-ray diffraction analyses classified *S. c. ricini* native fiber under group 3a, that is, they have a poly-l-alanine (PA) type *β*-sheet structure with an inter-sheet packing of 10.6 Å [5]. With the advancement of sequencing technology, the primary structure of *S. c. ricini* SF has been clarified to typically comprise of alternating repeat PA, Ala _(12-13)_, and Gly-rich sequence regions [1]. In contrast, that of the mulberry silkworm, *Bombyx mori* (*B. mori*) SF, mainly consists of a (GAGAGS)_n_ repeat sequence [6,7]. This characteristic assembly in the primary structure of *S. c. ricini* SF is almost similar to that of SF derived from silks produced by other *Saturniidae* wild silkworms such as *Antheraea pernyi* (*A. pernyi*) [8,9], *Antheraea yamamai* (*A. yamamai*) [10], *Antheraea assama* (*A. assama*) [11] and *Antheraea mylitta* (*A. mylitta*) [12], as well as the major ampullate (MA) silk of spiders (although their PA sequences are shorter, Ala_(5-6)_) [13,14]. Similar to the PA sequences in *S. c. ricini* and other native wild SF, X-ray diffraction, and infrared, Raman and solid-state NMR spectroscopy studies [15,16,17,18,19,20,21,22,23,24] have shown the (GAGAGS)_n_ repeat sequence regions in *B. mori* silk SF to primarily contribute to the formation of *β*-sheets [17,19,20,21,25,26]. In addition, the two kinds of SF have other structures such as *α*-helix and random coil [21,27].

The heterogeneous nature of SF is believed to influence the fiber structure and mechanical performance of silks. Consequently, variability in mechanical properties of silks has been closely linked to the differences in primary, secondary and hierarchical structures of SF [21,28,29]. For instance, *B. mori* and *S. c. ricini* silks have *β*-sheet structures but exhibit distinct mechanical properties; *S. c. ricini* native fiber exhibit a characteristic plateau region in their stress-strain curve unlike that from *B. mori* that show a distinct yielding point [21,28,29,30]. This unique feature, also observed in other wild silks [21,29], is often interpreted to be a result of some structural transitions, such as helical to zigzag conformations in poly(ethylene oxide) [31], *α*-helix to *β*-sheet conformations in keratin [32,33], and amorphous to crystalline states in natural rubber [34]. Since the predominant feature in saturniid fibroin sequences is the highly repetitive PA and Gly-rich regions, the phenomenon was also recently hypothesized to reflect co-ordinated molecular movement that may arise from the high degree of regularity [29].

Studies on the structural formation of *S. c. ricini* silk have widely been performed using silk dope from the posterior silk glands (PSG) of mature larvae. Based on ^13^C solid-state NMR spectroscopy, a film cast from liquid fibroin (LF) solution has been clarified to have a *α*-helical conformation [22,35,36], the amino acid component in the *α*-helix being dominantly from the PA regions [37]. The structural transition from *α*-helix to *β*-sheet during stretching of semi-dried silk dope [16], considered to correspond to the natural spinning process [22], was traced by ^13^C CP-MAS, X-ray diffraction and Raman spectroscopic analyses [16]. Further, Asakura et al. revealed that some amount of the *α*-helical conformation remains even in the native *S. c. ricini* fiber [38]. However, the aggregation state of the *α*-helices in the native fiber is yet to be clarified.

**Scheme 1:**
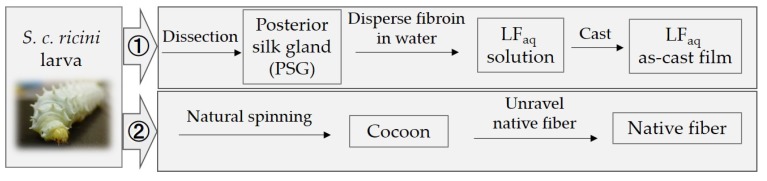
A summary of the schemes used in preparation of the *S. c. ricini* LF_aq_ as-cast film (route ①) and native fiber (route ②) samples.

In this study, we try to clarify (a) the aggregation state of residual *α*-helices in *S. c. ricini* native fiber, (b) whether they transform to *β*-sheets during stretching, (c) the effect on the physical properties of the fiber if the structural transformation occurs, and (d) if complete transformation of the residual *α*-helices can be attained. To achieve these, two kinds of samples were prepared as summarized in Scheme 1. (1). Silk dope was obtained from the PSG of mature larvae and diluted to give an aqueous solution of LF from which cast films were prepared (herein referred to as LF_aq_ as-cast films; refer to Section 3.1). (2). Native fibers were obtained from cocoons spun by larvae from the same batch from which the silk dope was obtained. Detailed comparative structural analyses of the silk samples were carried out by ^13^C CP-MAS solid-state NMR spectroscopy, Fourier transform infra-red spectroscopy (FTIR), wide angle X-ray diffraction (WAXD), thermal gravimetry (TG), and differential scanning calorimetry (DSC). Our results revealed that the residual α-helices in *S. c. ricini* native silk aggregate with a hexagonal packing and their strong contribution to the characteristic fiber physical properties was clarified.

## 2. Results and Discussion

### 2.1. Ordered α-Helix Structure in S. c. ricini Liquid Fibroin As-Cast Film

**Figure 1:**
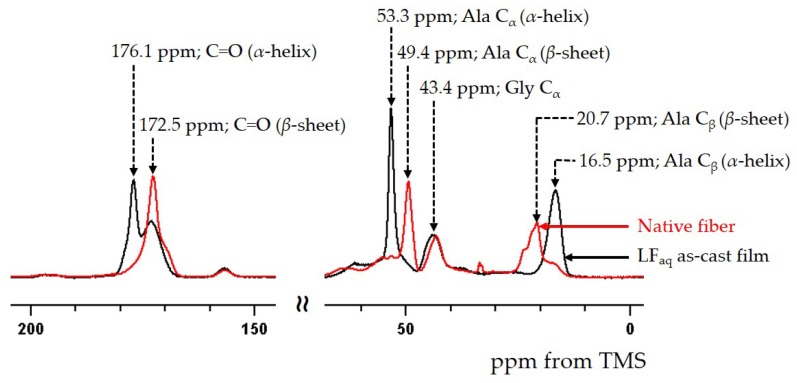
^13^C CP-MAS solid-state NMR spectra of *S. c. ricini* LF_aq_ as-cast film (spectrum in black) and native silk fiber (spectrum in red) from 0 to 60, and 150 to 200 ppm.

**Figure 2:**
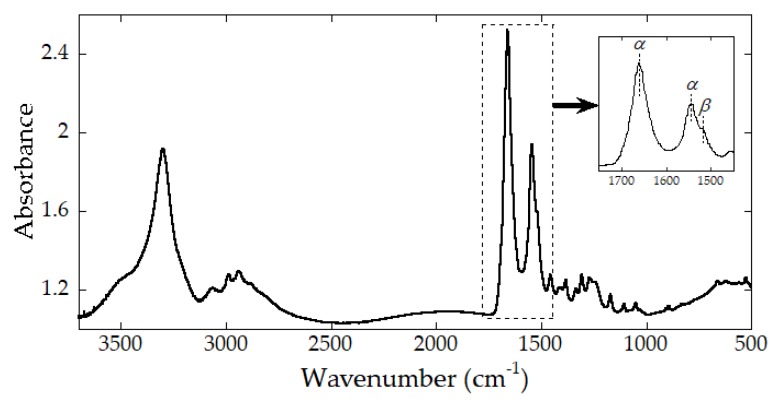
A representative FTIR spectrum of *S. c. ricini* LF_aq_ as-cast film. Inset is the spectrum showing the vibrational bands in the amide I and II regions.

Silk dope collected from the PSG of mature *S. c. ricini* larvae was diluted with milli-Q water to give a transparent aqueous solution of LF (LF_aq_). Films cast from this solution (LF_aq_ as-cast films) at 23 ± 2 °C and a relative humidity of 30 ± 2%, were transparent and flexible. Secondary structure of bio-polymers obtained over a broad spectral range may be handy in understanding and predicting their properties and potential applications [39]. However, in the current study, we focus on the aggregation state of the *α*-helical domains in the LF_aq_ as-cast film and native fiber, and clarification of such spectral features remains an important and interesting topic for future studies [40]. ^13^C CP-MAS NMR spectra of the LF_aq_ as-cast film and native fiber are shown in Figure 1. The chemical shift assignments for the Ala C_β_, Gly C_α_, Ala C_α_ and C=O spectral regions used in the current report are indicated in the spectra [16,21,24]. The native silk fiber was *β*-sheet-rich whereas the LF_aq_ as-cast film was *α*-helix-rich. These results are consistent with previous work [12,22], since *S. c. ricini* LF is known to have a well-structured *α*-helical conformation in the PA region [27]. This result was well supported by FTIR as shown in the typically *α*-helix-rich FTIR spectrum of the LF_aq_ as-cast film in Figure 2. The amide I region of the spectrum is assigned to the *α*-helical conformation whereas, the amide II region seems to contain a small amount of *β*-sheet or other components.

**Figure 3:**
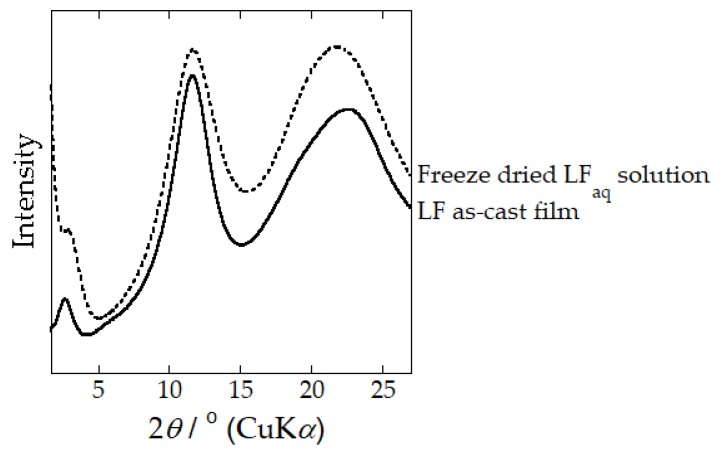
Wide angle X-ray diffraction (WAXD) 2*θ*-profiles obtained for *S. c. ricini* LF_aq_ as-cast film (solid line profile) and a freeze-dried sample of the LF solution from which the film was cast (dotted line profile).

A WAXD 2*θ*-profile of the LF_aq_ as-cast film is shown in Figure 3. A strong and sharp peak at 2*θ* = 11.6° (corresponding to *d* = 7.6 Å), a broad peak having an undefined peak-top at around 2*θ* = 22.7° (corresponding to *d* = 3.9 Å) as well as a distinct small-angle scattering peak at 2*θ* = 2.7° (corresponding to *d* = 32.7 Å) were detected. Similar peak combinations of the former two were observed for dried LF dopes of *S. c. ricini* [16] and *A. pernyi* [35,36] and assigned to the *α*-helix structure. Based on X-ray structural analysis of a highly-oriented crystalline *α*-helix sample of PA, Bamford et al. determined a hexagonal unit cell (*a* = 8.55 Å), giving a strong diffraction of the (10-10) plane at *d*_10-10_ = 7.40 Å [41]. A slightly lager *d*-spacing of the (10-10) plane indicates a slightly larger hexagonal unit cell dimension, probably affected by side chains of amino acid residues neighboring the PA sequence regions. The unclear broad peak at around 3.9 Å as well as the other similar observation in the abovementioned work on dried LF dopes from *S. c. ricini* and *A. pernyi* strongly suggest that the *α*-helical domains are aggregated with a hexagonal packing, but not perfectly crystallized as observed in PA [41] and poly-*γ*-methyl-l-glutamate [42]. The ordered aggregation size along the (10-10) lattice direction (roughly corresponding to the diameter of the cross-sectional area of the aggregated *α*-helices bundle) was evaluated from the peak width of the (10-10) peak using the Scherrer equation to be 32 Å. This dimension is well consistent with the small-angle scattering corresponding to a periodicity of 32.7 Å, suggesting that the aggregated *α*-helices bundles further form a hierarchical super-array with a neighboring distance of around 32–33 Å.

From the X-ray analyses, it is evident that the fibroin molecules in the LF_aq_ as-cast film assemble into an ordered aggregated structure. To clarify the stage during which structural formation of the ordered *α*-helices occurs, WAXD analysis of a freeze-dried sample, frozen at −80 °C, of the LF-solution from which the LF_aq_ film was cast, was performed. The WAXD 2*θ*-profile of the freeze-dried sample, shown in Figure 3, was essentially similar to that of the LF_aq_ as-cast film. Considering previous work showing successful immobilization of the structure of SF by freezing at −80 °C [43,44], our experimental result strongly indicates the existence of the ordered aggregated structure in the aqueous state. This is consistent with a common consensus in natural spinning of spider silks [45] and *B. mori* silkworm silk [46,47], in which LF comes out of the spinneret in a liquid crystalline or nematic state. The formation of an ordered aggregation state in the *α*-helical domains in the solution state of *S. c. ricini* seems quite reasonable to achieve efficient natural spinning, during which highly oriented and crystalline fiber is produced with consumption of minimum energy.

**Figure 4:**
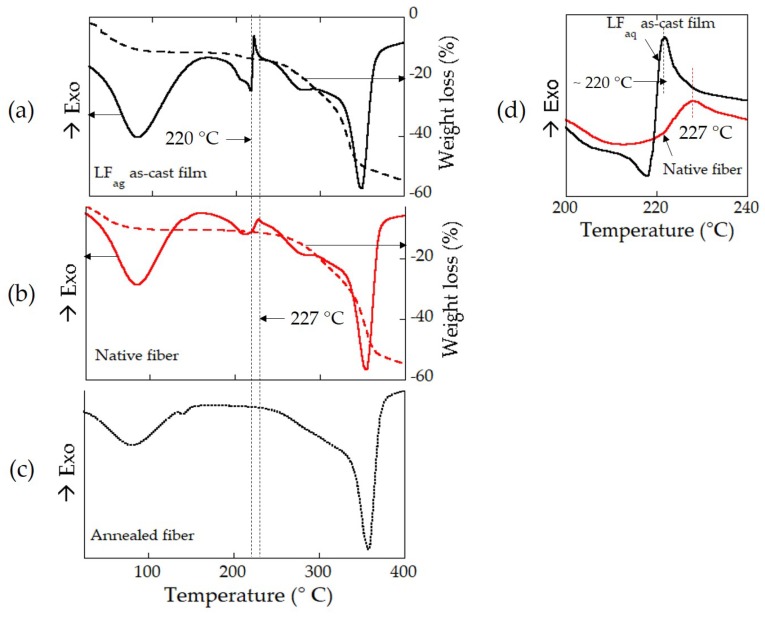
Thermal gravimetry (TG) and differential scanning calorimetry (DSC) profiles of *S. c. ricini* LF_aq_ as-cast film (**a**), and native (**b**), and heat-treated (220 °C, 1 min; **c**) fibers. Panel **d** shows a comparison of the LF_aq_ as-cast film (profile in black) and native silk fiber (profile in red) DSC profiles between 200 and 240 °C.

Figure 4 shows the TG and DSC profiles of the LF_aq_ as-cast film, and native and heat-treated *S. c. ricini* silk fiber. As seen in Figure 4a,d, a remarkably sharp exothermic peak, speculated to correspond to a structural transition from the ordered aggregated *α*-helix to *β*-sheet structure, was detected at ~220 °C in the DSC profile of the LF_aq_ as-cast film.

**Figure 5:**
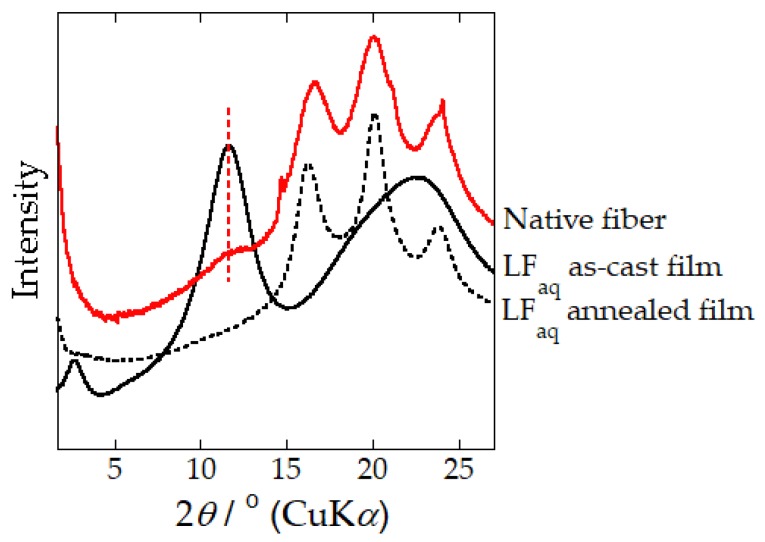
WAXD 2*θ*-profiles of native *S. c. ricini* silk fiber (profile in red), and as-cast (profile in black solid line) and heat-treated (220 °C, 1 min; profile in black dotted line) LF_aq_ cast films.

To clarify the kind of transition that occurs, as-cast and heat-treated (220 °C, 1 min) LF_aq_ films were subjected to WAXD analysis. WAXD 2*θ*-profiles of native fibers (same profile shown in Figure 3), and the as-cast and heat-treated LF_aq_ film are shown in Figure 5. A clear transition from the ordered aggregated *α*-helix to *β*-sheet structure, induced by heat treatment, was confirmed. Such a narrow temperature window in the structural transition in the LF_aq_ as-cast film (Figure 4a,d) is considered to result from instantaneous solid-to-solid phase transition from the hexagonally packed *α*-helix to the *β*-sheet structure with minimum molecular movement.

### 2.2. Ordered α-Helix Structure in S. c. ricini Native Silk Fiber

Although the WAXD *β*-sheet profile of the heat-treated LF_aq_ cast film was essentially similar to that of the typically *β*-sheet-rich native *S. c. ricini* silk fiber (Figure 5), a small but highly reproducible difference existed: A shoulder peak in the native silk fiber profile (indicated by the dotted red line) corresponding to the peak assigned to the ordered aggregated *α*-helix structure in the LF_aq_ as-cast film in the preceding section was detected. We speculated the peak to indicate the presence of a small amount of residual ordered aggregated *α*-helix structure in the native fiber. This speculation was supported by the DSC results of native fiber in which a peak similar to the characteristic sharp exothermic peak in the DSC profile of LF_aq_ as-cast film was detected (Figure 4b). However, in the DSC profile of native fiber, the exothermic peak was broad and had a slightly higher peak top temperature (~227 °C). Moreover, the endothermic peak was reversed, that is, it appeared at a slightly lower temperature (Figure 4d).

To account for the residual ordered *α*-helices detected in the native silk fiber, it is considered that aggregated domains with higher ordering level preferentially transform to *β*-sheets during natural spinning, whereas those with a lower ordering level are not. In other words, the ordered aggregated *α*-helix in native *S. c. ricini* fiber is considered to be partially collapsed and has a slightly unstable structure. Hence, the structural transition (endothermic reaction) starts at a slightly lower temperature in the fiber than in the as-cast film. Often, the *β*-sheet structure is difficult to form, hence, subsequent *β*-sheet formation does not occur until a higher temperature is attained. For instance, in the assembled portion of amino acids where side chains are bulky, larger molecular motion (higher energy) is required because they hinder *β*-sheet formation, hence the exothermic peak occurs at a higher temperature. This implies that, sites where *β*-sheet formation is relatively difficult that are untransformed during native spinning exist in the ordered aggregated *α*-helix structure. Further, partial collapse of the originally ordered structure that results in broadening of the transition peak in native silk fiber is considered to result from stretching and compression during spinning. The presence of the structure that does not easily transform to *β*-sheet may be considered intentional and a strategy of the *S. c. ricini* silkworms to impart flexibility in the silk.

**Figure 6:**
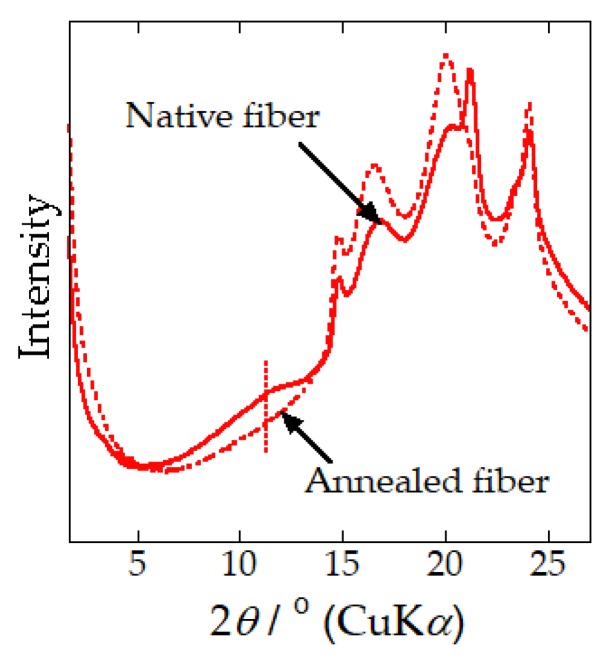
WAXD 2*θ*-profiles of native (profile in red solid line) and heat-treated (220 °C, 1 min; profile in red dotted line) *S. c. ricini* fibers.

To clarify if the peak could not be ascribed to *β*-sheet but the ordered aggregated *α*-helix structure, native silk fibers were heat-treated as previously discussed for the as-cast LF_aq_ film and subjected to WAXD and DSC analyses. WAXD 2*θ*-profiles of native (same profile shown in Figure 5) and heat-treated silk fibers are shown in Figure 6. Consistent with our earlier results for the heat-treated LF_aq_ film, the peak disappeared accompanied by enhancement of the intensity of the peaks assigned to *β*-sheet structure. Similar disappearance of the exothermic peak in the DSC profile for heat-treated fibers was observed (Figure 4c). These results confirm that though low in abundance, native *S. c. ricini* silk fibers contain an ordered aggregated *α*-helix structure.

### 2.3. Impact of Heat-Treatment on the Mechanical Properties of Single S. c. ricini Native Fiber

In the preceding section, we successfully detected a signal peak of residual ordered aggregation of *α*-helices in the WAXD pattern of *S. c. ricini* native silk fiber. The residual helices were confirmed to transform into *β*-sheet due to heat-treatment at around 220 °C by WAXD and DSC. Moreover, it is well established that stretching induces *α*-helix to *β*-sheet structural transition in various types of silks including *S. c. ricini* LF-based materials [16,18], spider [48], and regenerated *B. mori* [47] silk. However, the relationship between this structural transition and the characteristic plateau deformation behaviour in the stress-strain curve of *S. c. ricini* native fibers remains unclear. To clarify the relationship, the effect of heat-treatment on the plateau phase in the stress-strain curve of single *S. c. ricini* native silk fibers was investigated. The cross-sectional areas of native silk fiber has been shown to vary along the silk fiber axis [29], due to flaws possibly associated with the larvae’s spinning behaviour [49] often affected by among other factors, temperature and relative humidity [50]. Hence, since the cross-sectional areas of the individual test fibers were not evaluated in the current study, fiber sampled from adjacent portions of single fiber carefully unravelled from the middle layer of the middle part of the cocoon were used for the tensile tests before and after heat-treatment, respectively, to reduce variability. Therefore, the mechanical properties discussed here are an estimation from the representantive force (N) against strain (%) plots. Further, the heat-treatment temperature was chosen based on the DSC data discussed in preceding section, where *α* to *β*-sheet structural transition was confirmed, under a similar heat-treatment regime for native fiber (cocoon sections) used for WAXD analyses.

**Figure 7:**
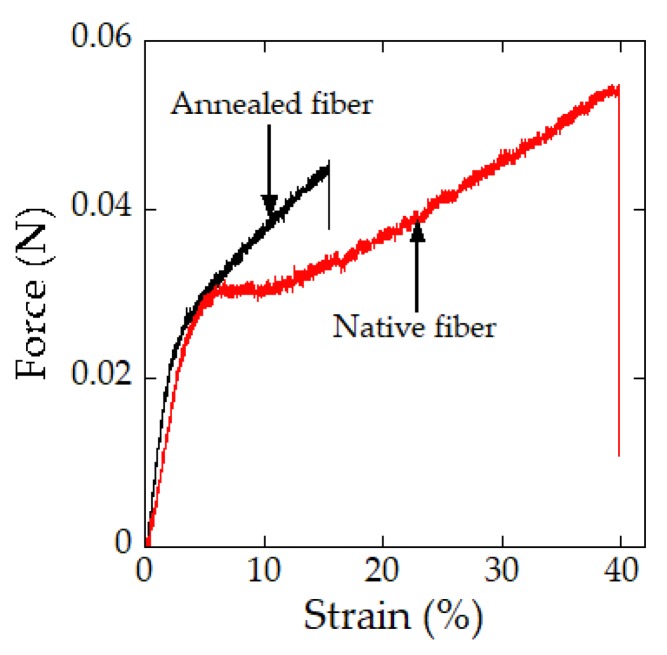
Representative force-strain curves of native (curve in red), and heat-treated (220 °C, 1 min; curve in black) *S. c. ricini* single silk fibers.

The results of the tensile tests of native and heat-treated *S. c. ricini* silk single fibers were reproducible, and representative stress-strain curves are shown in Figure 7. As expected, a distinct plateau region was detected in the stress-strain curve of native silk fiber, but was absent or significantly diminished in the stress-strain curve of the heat-treated fiber. Further, the stress-strain curve of the heat-treated single fibers transformed to be like that of *B. mori* and exhibited a lower strain to break (extensibility). The disappearance of the plateau phase and lower extensibility were speculated to be due to *α* to *β*-sheet structural transition, caused by the heat-treatment (see Figure 7), already clarified in the preceding section. This is because heat-treatment reduces the residual *α*-helices (increases the *β*-sheet content) in the fibers which often results in enhanced stiffness [51]. In the current study, the fiber spinning process has been shown to play a key role in determining the unique material properties of *S. c. ricini* native fibers, in addition to the highly repetitive SF protein amino acid sequence in various silks recently reported by Malay et al. [29].

### 2.4. Strain-Dependent α-Helix to β-Sheet Transition in S. c. ricini Native Silk Fiber Traced by WAXD

**Figure 8:**
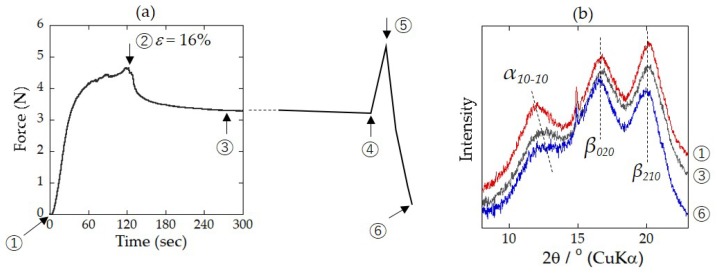
(**a**) Force-time curve, and (**b**) WAXD 2*θ*-profiles of a parallel-aligned bundle of *S. c. ricini* native fiber before stretching ① (profile in red), just after the plateau region (*ε* = 16%; ③; profile in black), and after the fiber bundle broke ⑥ (profile in blue).

To investigate the relationship between the plateau deformation behavior and the residual *α*-helix to *β*-sheet structural transition, WAXD measurements of a parallel-aligned bundle of *S. c. ricini* native fibers were performed at three different stages; before stretching (intact fiber-bundle; ①), just after the plateau region ③, and after fiber bundle breaks ⑥. As depicted in Figure 8a, stretching was paused just after the plateau region, corresponding to *ε* = 16% (indicated by arrow ②). This was immediately followed by a rapid drop in force, corresponding to stress relaxation, as seen beyond point ②. The second WAXD measurement was started after the stress relaxation was almost saturated as indicated by arrow ③. Due to inter-strand hydrogen bonding, *β*-sheets are energetically favorable, that is, they are high-temperature relaxed structures [52] and the structural transition from *α*-helix to *β*-sheet is irreversible. Hence, the influence of stress relaxation on the purpose of this WAXD measurement, that is, to trace the changes of the signal peak of ordered aggregation of *α*-helices, was considered negligible.

After the second WAXD measurement, the fiber bundle was again stretched (indicated by arrow ④) until it broke (indicated by arrows ⑤ and ⑥). This was followed by a third WAXD measurement ⑥, without distorting the broken but parallel-aligned fiber bundle on the Linkam tensile stage. The equatorial 2*θ*-profiles scanned from the resultant WAXD patterns were as shown in Figure 8b. In addition to the equatorial (020) and (210) *β*-sheet reflections, the signal reflection of the (10-10) ordered residual *α*-helices was clearly detected in intact native fiber. This peak was observed to decrease gradually with increasing strain. A significant decrease in the peak intensity of this signal reflection was observed after the fiber bundle broke; however, the decrease majorly occurred before the end of the plateau region. This observation suggests *β*-sheet formation to occur at the expense of the *α*-helix structure.

## 3. Experimental Section

### 3.1. Preparation of S. c. ricini LF_aq_ As-Cast Film and Native Fiber

*S. c. ricini* silkworm larvae were reared in the laboratory on an all-instar artificial diet, SilkMate L4M (Nosan Corp., Yokohama, Japan) at 25 ± 3 °C and a relative humidity of 65 ± 3%. Late 5th instar larvae, just after gut purging, were anaesthized in ice for 15 min and dissected to obtain intact silk glands. The silk glands were briefly rinsed in cold Milli-Q water (~4 °C) and silk dope in the lumen of the PSG was drained into cold Milli-Q water and carefully rinsed by exchanging the Milli-Q water several times to remove any contaminants. The thus-obtained silk dope was diluted with Milli-Q water to give an aqueous solution of fibroin, herein referred to as liquid fibroin (LF_aq_), that was gently cast on polystyrene Petri-dishes. After drying at 23 ± 2 °C and a relative humidity of 30 ± 2% [46], transparent and flexible films that could be peeled off the substrate with negligible mechanical damage were obtained. The thickness of the cast films was controlled by adjusting the concentration or volume of LF cast.

For comparison, some *S. c. ricini* larvae from each batch reared (from which the LF used in the preparation of the as-cast films was obtained) were let to spin cocoons whence native fibers analysed was obtained as described in the subsequent sections.

### 3.2. ^13^C Cross Polarization–Magic Angle Spinning (CP/MAS) Solid-State NMR Spectroscopy

^13^C CP/MAS solid-state NMR measurements were performed with a Bruker Avance 600 WB (Karlsruhe, Germany) spectrometer with a magnetic field of 14.1 T at room temperature. The spectrometers were operated at a ^13^C NMR frequency of 150.94 MHz. For each measurent, the fibroin protein sample was cut into small pieces, packed into a solid-state probe, and spun at a MAS frequency of 10.0 kHz in a 4.0 mm Ø zirconia rotor sample tube. A ^1^H 90° pulse length of 3.5 *μ*s and ^1^H-^13^C cross-polarization (CP) contact of 70 kHz were employed for the CP experiments. High-power ^1^H decoupling using the SPINAL-64 method was employed. The repetition time for the CP experiments was 3.0 s and the ^13^C chemical shifts were calibrated externally through the adamantane methylene peak at 29.5 ppm relative to TMS at 0 ppm. For native fiber, cocoon sections analysed were sampled from the middle section of the middle layer of the cocoons obtained in sub-Section 3.1.

### 3.3. Fourier Transform Infrared (FTIR) Spectroscopy

Since amide vibrations depend on the secondary structure of proteins, FTIR spectroscopy is often used in studying the secondary structures of proteins. In the current study, FTIR measurements of the LF_aq_ as-cast films were recorded at room temperature with an FTIR-620 (JASCO International Co. Ltd., Hachioji-shi, Japan) spectrometer in the transmission mode. For each measurement, 32 scans were co-added in the spectral range 4000 to 400 cm^−1^ with a 2 cm^−1^ resolution. The resultant spectrum was corrected by subtracting a background spectrum recorded under similar scan conditions. The assignments of conformation sensitive bands for *S. c. ricini* SF used in this report were: ~1625 and ~1519 cm^−1^ as *β*-sheet structure [21,53] and ~1658 and 1545–1548 cm^−1^ as *α*-helix [12,53].

### 3.4. Wide-Angle X-ray Diffraction Analyses

To clarify the aggregation state of the *α*-helical domains in the LF_aq_ as-cast film and native fiber, WAXD measurements were carried out with a 3.5 m NANOPIX X-ray diffractometer (Rigaku Corp., Tokyo, Japan) (40 kV, 30 mA, Cu−K_α_ radiation (λ = 1.5418 Å)) equipped with a highly sensitive single-photon counting pixel HyPix-6000 2D detector with a pixel size of 100 × 100 µm^2^ (Rigaku Corp., Tokyo, Japan) as earlier described [54]. Calibration of the camera distance was carried out using cerium oxide powder and a background diffraction pattern collected under conditions similar to those used for sample analysis was subtracted from each measurement. To obtain the WAXD 2*θ*-profile of LF, an aliquot of the solution was rapidly frozen to −80 °C in an ultra-low temperature Nihon freezer (NF-10SF3, Tokyo, Japan) and lyophilized in an EYELA vacuum freeze-dryer (FDU 830, Tokyo, Japan) for 24 h. The crystallite sizes were evaluated on the basis of the Scherrer equation. For the heat-treated LF_aq_ film and native fiber (sampled from the middle layer of the middle section of cocoons obtained in sub-Section 3.1), the samples were cut into small pieces each about 0.5 × 0.5 mm and separately sandwiched between thin glass slides (~1.0 mm thick), alongside a thin and sensitive thermocouple to monitor the sample temperature directly. These were set on a digital hot-plate (Corning PC-420D), heated to 220 °C, and kept at this temperature (annealed) for about 1 min and allowed to cool to room temperature.

### 3.5. Thermal Analyses

Differential scanning calorimetry (DSC) measurements were performed with a DSC Q200 analysis system (TA Instruments, New Castle, DE, USA). Instrument calibration for heat flow and temperature were done using indium, whereas for heat capacity, aluminum and sapphire were used. For each measurement, about 5.0 mg of the fibroin protein sample was encapsulated in an aluminum pan and heated between −30 and 430 °C at 2 °C min^−1^ under a dry nitrogen gas flow of 50 mL min^−1^.

The concentrations of LF, and the weight loss profiles of the LF_aq_ as-cast film and native fiber were determined by thermal gravimetry (TGA) with a Thermoplus TG 8120 system (Rigaku Corp., Tokyo, Japan). For each measurement, about 1.0 mg of the fibroin protein was heated in an aluminium pan from room temperature to 430 °C at a heating rate of 2 °C min^−1^ under a dry nitrogen gas flow of 200 mL min^−1^.

### 3.6. Tensile Tests of Single S. c. ricini Silk Fibers

Tensile properties of *S. c. ricini* single fibers were measured using an EZ Test/CE mechanical stage (Shimadzu Co., Kyoto, Japan) equipped with a 5 N load cell. All tests were carried out in the dry state at 23 ± 2 °C, and a relative humidity of 37 ± 2%. Single fibers, each approximately 6.0 cm, were carefully unravelled from the middle section of the middle layer of the native cocoons obtained in sub-Section 3.1, sub-divided and labelled systematically (say A and B, for native and heat-treated tests, respectively). One set of the fibers were set using a kapton tape and sandwiched between thin glass slides (~1.0 mm thick), alongside a thin and sensitive thermocouple and heat-treated as described in sub-Section 3.4. Each fiber (native and heat-treated) was then mounted without pre-load onto rectangular paper frames with an initial gauge length of 15 mm and their tensile deformation behaviour was evaluated at a cross-head speed of 2 mm min^−1^. The tensile properties reported were averaged from three measurements, each for native and heat-treated fiber. A paired *t*-test using GraphPadPrism 5 (GraphPad Software, Inc., San Diego, CA, USA) was used to compare native and heat-treated data at 95% confidence interval.

### 3.7. Strain-Dependent Structure Transformation Monitored by WAXD

To monitor structural changes in *S. c. ricini* native fiber during tensile deformation, a parallel-aligned bundle, with a thickness of about 0.5 mm, was carefully prepared from single fibers obtained from the cocoons as already described [54]. The fiber bundle was fixed on a mechanical tensile stage (Linkam Scientific Instruments, Epsom, Tadworth, UK) with a 3.0 mmØ hole for the X-ray beam to pass through. The micro-stretcher was equipped with a 10 N load cell and coupled to the 3.5 m NANOPIX X-ray diffractometer (Rigaku Corp., Tokyo, Japan) (see sub-Section 3.4). The gauge distance was 20 mm and tensile deformation of the fiber bundle was carried out at a cross-head speed of 2 µm sec^−1^. WAXD measurements were carried out at three stages, that is, before stretching, just after the plateau region, and after the fiber bundle breaks. The duration of exposure for each WAXD measurement was 30 min.

## 4. Conclusions

The broad peak at around 3.9 Å in the 2*θ*-profiles of the LF_aq_ as-cast film and native fibers revealed that the residual *α*-helical domains in the otherwise typically *β*-sheet-rich native fiber are aggregated in a hexagonal packing. X-ray scattering and DSC revealed transformation of the residual *α*-helices to *β*-sheets due to heat-treatment at ~220 °C. The structural transition was accompanied by disappearance of the characteristic plateau region in the force-strain curve of the native fibers. Further, based on WAXD results before and after tensile deformation of the native fiber, we successfully elucidated the structural change occurring in the plateau region to be *α*-helix to *β*-sheet. Our findings clearly demonstrate the fiber formation process (natural spinning) to be critical in determining the physical properties of native *S. c. ricini* fibers, that is, the larvae by design keep some *α*-helices untransformed to impart flexibility in the native silk.
